# Comparison of Microleakage and Thickness of Resin Cement in Ceramic Inlays with Various Temperatures

**DOI:** 10.5681/joddd.2014.008

**Published:** 2014-03-05

**Authors:** Homayoun Alaghemand, Faezeh Abolghasemzadeh, Farzaneh Pakdel, Reza Judi Chelan

**Affiliations:** ^1^Dental Material Research Center, Associate Professor, Department of Operative Dentistry, Faculty of Dentistry, Babol University of Medical Sciences, Babol, Iran; ^2^Assistant Professor, Department of Operative Dentistry, Faculty of Dentistry, Babol University of Medical Sciences, Babol, Iran; ^3^Assistant Professor, Department of Oral Medicine, Faculty of Dentistry, Tabriz University of Medical Sciences, Tabriz, Iran; ^4^DentalMaterial Research Center, Member of Research Committee of Medical Students, Student of dentistry , Faculty of Dentistry , Babol University of Medical Sciences, Babol, Iran.

**Keywords:** CAD-CAM, ceramic, film thickness,, microleakage

## Abstract

***Background and aims.*** Microleakage is still one of the major problems of composite-based restorations.This study compared the microleakage and thickness of resin cement in ceramic inlays with various temperatures.

***Materials and methods.*** Class V cavities were prepared on the buccal and lingual aspects of thirty human molars with occlusal margins in enamel and gingival margins in dentin (3 mm wide, 5 mm long and 2 mm deep). Laboratory-made inlays (LMI) were used for buccal cavities, and CAD/CAM inlays (CMI) were used for lingual cavities. All the cavities were divided into six groups (n=10): 1) LMI at -5°C; 2) LMI at 50°C; 3) LMI at room temperature (25°C); 4) CMI at -5°C; 5) CMI at 50°C; 6) CMI at room temperature (25°C). Inlays were bonded to cavities in a pulp pressure- and temperature-simulating device. After thermocycling and dye penetration, the teeth were divided into two mesiodistal halves. Amount of dye penetration and film thickness were measured under a stereomicroscope and analyzed with Kruskal-Wallis, Wilcoxon and Spearman's correlation tests ( = 0.05).

***Results.*** There were no statistically significant differences in leakage between different inlay temperatures (P > 0.05). The mean cement thickness in laboratory-made inlays (gingival margin, 83.7 ± 11 and occlusal margin, 84.7 ± 19) was greater than that in CAD/CAM inlays (gingival margin, 69 ± 16 and occlusal margin, 84.7 ± 16). No correlation was found be-tween cement thickness and microleakage either in enamel or dentin for any of the ceramic systems.

***Conclusion. ***Differences in inlay temperature had no effect on microleakage. CAD/CAM inlays had lower cement thickness than laboratory-made inlays, but this was not related to their microleakage.

## Introduction

By using porcelain as a restorative material, a new era began in cosmetic dentistry. Ceramic ls have now become the bedrock of cosmetic dentistry. Ceramic inlays have less leakage and seat better than composite inlays. Bond strength of resin cements to etched ceramic inlays is stronger and more durable than composite inlays. Resin cement is the only recommended material for cementation of ceramic inlays. This cement restricts microleakage, increases the strength of the restoration and in short term reinforces residual tooth structure.^1^

In recent years, many improvements have been made in mechanical and physical properties of composite resins, but microleakage is still one of the major problems of composite-based restorations. It seems using composite resins without polymerization shrinkage is the main key to placement of a leakage-free restoration.^2^

Nowadays, it has been proven that composite's flow at the free surface can reduce the shrinkage stress of composite in bonded surface and reduce restoration microleakage. Reducing the rate of polymerization provides more time for composite to flow at free surface and reduce the shrinkage stress. Temperature of composite resin during setting is one of the factors that influences the amount of polymerization shrinkage stress. Low temperature causes reduction in the degree of conversion and polymerization rate. In addition, thermal expansion of cooled composite resin when placed under the oral cavity temperature can compensate polymerization shrinkage to some extent.^3^

Many studies have investigated the effect of temperature on microleakage of direct composite resin restorations,^4-6^ but none of them paid attention to the effect of different temperatures of ceramic inlays on microleakage of room-temperature resin cement.

Since the amount of film thickness was different in the laboratory-made and CAD/CAM inlays,^7,8^ we used both types of restoration to evaluate the effect of film thickness on microleakage of resin cement. Therefore, the aim of this in vitro study was to compare the microleakage and thickness of resin cement in ceramic inlays with various temperatures. The null hypothesis was that temperature of ceramic inlay and cement thickness would not affect microleakage.

## Materials and Methods

Thirty caries-free freshly extracted human molars stored in saline solution were selected for this study. For disinfection, the teeth were immersed in 0.05% thymol solution for no longer than 6 months.^9^ Standardized Cl V cavities (5 mm in length, 3 mm in width, and 2 mm in depth) were prepared on the buccal and lingual aspects of each tooth, with the gingival wall extending beyond cemento-enamel junction, while the occlusal walls extended to the enamel. An impression was made from the buccal cavities with additional silicone material (Elite HD+, Zhermack, Rovigo, Italy) and sintered feldspathic porcelain inlays (Ceramco, Dentsply, USA) were made for each cavity according to manufacturer’s instructions. CAD/CAM (CEREC3D, Sirona Dental Systems, Germany) feldspathic inlays (CEREC® Blocs, Sirona Dental Systems, LLC, Germany) were made for lingual cavities. The cavities were divided into six groups (n=10):

1. Sintered feldspathic inlays cooled to −5°C
2. CAD/CAM feldspathic inlays cooled to −5°C
3. Sintered feldspathic inlays warmed to 50°C
4. CAD/CAM feldspathic inlays warmed to 50°C
5. Sintered feldspathic inlaysat room temperature (25°C)
6. CAD/CAM feldspathic inlays at room temperature (25°C)

### Pulp Pressure and Temperature Simulator 

**Figure 1.**
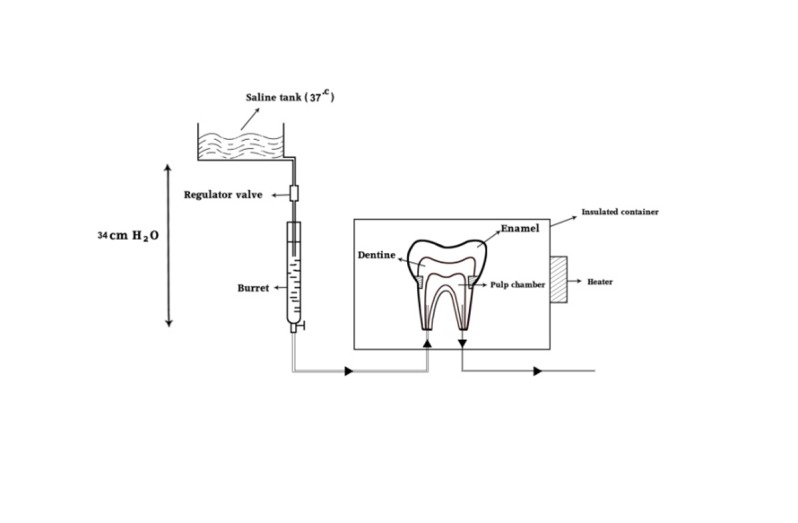
Pulp pressure and temperature simulator.

A device was made for simulation of oral conditions, which sent water (37°C) with 35 cmH_2_O pressure into the pulp chamber. Prior to the bonding process, air of the chamber was kept at 37°C. The device consisted of an insulated container in which inlays were bonded to the teeth. The chamber temperature was controlled by a heater and a temperature-regulating device (thermocouple) at the same temperature as the oral cavity (37°C). To simulate the pulpal pressure, a burette was used. The burette was connected to a normal saline tank (the normal saline temperature was kept at 37°C with a heater and thermocouple) and by a regulator valve; the water flow was adjusted so that the water height was 34–40 cm. From the end of the burette, a narrow pipe came to the insulated chamber and was connected to the apex of tooth root with an 18-gauge needle. The apex of each root was dilated so that the needle could enter and was connected to the apex; therefore, water could enter from a root to the pulp chamber and pass through the other root (Figure 1).

### Inlay Bonding Procedure 

The materials used in the bonding procedures are described in Table 1.

**Table 1:** Composition of materials used for this study according to manufacturer’s data

Material	Manufacturer	Type	Composition
Panavia F2.0	Kuraray Co.,	Dual-cure	ED Primer A: HEMA, 10-MDP, 5-NMSA
	Osaka, Japan	adhesive system	ED Primer B: 5-NMSA
			Paste A: 10-MDP, Bis-GMA, filler, benzoyl peroxide, photoinitiator Panavia F2.0 Osaka, Japan adhesive system Paste B: Bis-GMA, filler, Sodium fluoride, amine
Margin Bond	Coltene/Whaledent, Switzerland	Unfilled resin	BISGMA:35-40 %
			TEGDMA: 20-25 %
Silane	Ultradent Dental Co, South Jordan, Utah, USA	Porcelain primer	Methacryloxy propyltrimethoxy silane:5-15% Ethanol:87%
Hydrofluoric Acid	ultradent Dental Co, South Jordan, Utah, USA	Porcelain etchant	Hydrofluoric Acid: 9.5%
HEMA: 2-Hydroxyethyl methacrylate			
10-MDP: 10-Methacryloyloxy decyl dihydrogenphosphate			
5-NMSA: N-methacryloyl 5-aminosalicylic acid			
Bis-GMA: Bisphenol glycidyl methacrylate			
TEDGMA: Triethylene glycol dimethacrylate			

The inlays were etched for 90 seconds with 9.5% hydrofluoric acid (HF) (Ultradent Dental Co, South Jordan, Utah, USA) followed by rinsing with water for 30 seconds and drying with an air stream. One-bottle silane coupling agent (Ultradent Dental Co, South Jordan, Utah, USA) was applied over the etched region. The inlays were placed in a freezer at -5°C temperature at least for 5 minutes before the bonding process for cooling and were placed in a hot air oven at 50°C temperature at least for 5 minutes before the bonding process for warming. Panavia F2.0 resin cement (Kuraray Dental Co, Okayama, Japan) was used to bond inlays. One drop from each bottle of Panavia ED primer was mixed and applied to tooth surface as bonding agent according to manufacturer's instructions. Before bonding a thin layer of unfilled resin (Margin Bond, Coltene/Whaledent, Switzerland) was applied to inlays.^10^ The inlay was bonded to the tooth while the tooth was connected to the pulp pressure- and temperature-simulating device. Resin cement was cured for one minute with an LED light-curing device (Valo, Ultradent Dental Co, South Jordan, Utah, USA). The light intensity was 1000 mW/cm^2^.

###  Thermocycling

After the bonding procedure, the samples were thermocycled for 500 cycles^11^ at 5°C/55°C water baths with 20 seconds of dwell time and 10 seconds of transfer time for each.

### Microleakage Test and Cement Film Thickness Measurement 

After thermocycling, the apices of the teeth were sealed with composite resin. All the surfaces of the teeth, except for 1 mm around the margins of each restoration, were sealed with 2 coats of nail varnish. The teeth were immersed in 50 wt% silver nitrate for 2 hours in a dark place. Then the samples were rinsed under running water and immersed for 4 hours in developing solution under fluorescent light and then flushed under running water.^12^ Then the samples were mounted in epoxy resin and sectioned buccolingually from the middle of restorations. Each half was observed under a stereomicroscope (×40 magnifications) and scored for the degree of dye penetration at the occlusal and cervical walls:

0: No dye penetration

1: Dye penetration into the enamel or extending to half of the cervical wall

2: Dye penetration beyond the dentinoenamel junction or to more than half or the entire cervical wall

3: Dye penetration into the pulpal wall^13^

After taking digital images from the samples with a stereomicroscope (×40 magnification) (SZX12, Olympus America, Melville, NY, USA) the cement film thickness was measured with Analysis LL Starter software (Inet Soft Technology Corp., New Jersey, USA).

Data was analyzed by SPSS 18. Kruskal-Wallis test was used to compare leakage between different temperatures. Wilcoxon signed ranks test was used to compare the mean cement film thickness of enamel and dentinal margin of CAD/CAM and laboratory-made inlays and compare microleakage of dentinal and enamel margins of different types of inlays at different temperatures. Spearman's correlation coefficient was used to evaluate the relation between resin cement film thickness and microleakage at enamel and dentinal margins at all the temperatures. Statistical significance was set at 5% (P<0.05).

## Results

Table 2 shows the means of microleakage scores at occlusal and gingival margin of the samples. Kruskal-Wallis test did not reveal any significant differences in microleakage between the groups (P>0.05).

**Table 2:** Comparison of microleakage at occlusal and gingival margins between CAD/CAM and laboratory-made inlay groups

	Microleakage mean			
	-5°C	50°C	25°C	P
Gingival margin (LMI)	0	3	3	0.29
Gingival margin (CMI)	0	0	3	0.21
Occlusal margin (LMI)	0	0	3	0.83
Occlusal margin (CMI)	0	0	3	0.27
LMI: laboratory made inlays				

Wilcoxon test showed significant differences between the mean cement film thickness of laboratory and CAD/CAM inlays (P<0.05). The minimum cement film thickness belonged to CAD/CAM inlays (occlusal margin: 63.4±16, gingival margin: 69±16), and maximum cement film thickness belonged to laboratory inlays (occlusal margin: 84.7±19, gingival margin: 83.7±11).

Wilcoxon test showed that the difference in microleakage between gingival and marginal margins was statistically significant only in laboratory and CAD/CAM inlays at 25°C (P<0.05). The difference of mean microleakage was not statistically different at enamel margins between different temperatures (P>0.05). The difference of microleakage between gingival margins of laboratory inlays and CAD/CAM inlays was statistically significant only at 50°C and the difference of microleakage between the occlusal margins of laboratory inlays and CAD/CAM inlays was statistically significant only at -5°C.

Spearman’s correlation coefficient showed no significant relation between resin cement film thickness and microleakage at enamel and dentinal margins at all the temperatures (P<0.05).

## Discussion

In this study, there was a slight decrease in microleakage in low- and high-temperature groups but this relationship was not significant statistically.

Microleakage studies use different methods. In this study dye penetration method was used because of its simple procedure and easy and accurate ability of observation by digital imaging.^14^Several dye solutions are available for microleakage tests but silver nitrate dye penetration may be a particularly demanding test of marginal seal because silver ions are smaller than the bacteria that usually live in the oral cavity.^15^ Therefore, in this study silver nitrate dye was used.

Many studies have investigated the effect of different temperatures of composite on microleakage. Some studies have shown that cold insert causes reduction in composite resin microleakage.^2,16,17^ Asmussen^18^ found that the filling could be cooled through a certain temperature range without marginal gap being formed.

Other studies have shown less microleakage with warmed composite resin.^19,20^ dos Santos et al^4^ found that warm composite resin (54°C and 60°C) had no effect on microleakage when LED light-curing system was used, but with QTH light-curing systems they found less microleakage.

Some studies have shown that different temperatures have no effect on microleakage.^3,5,6,21^ Daronch et al^22^ found no significant difference between the microleakage of warmed composite resin at 140°F, room temperature composite resin and flowable composite resin except for enamel margin of class V cavities; at room temperature composite resin microleakage was less than that of flowable composite resin.

To date, no research study has evaluated the effect of cooling or heating ceramic restorations on resin cement microleakage; only Morais et al^23^ evaluated the effect of heating the resin cement and ceramic restorations on bond strength. Since cooling the resin increases cement viscosity, interfering with restoration seating, and warming could increase its viscosity, resulting in difficulties in cement handling, in this study inlays were warmed or cooled instead of the resin cement.

Different studies have used different temperatures for warming and cooling composite resins but the exact reason for selecting these temperatures has not been mentioned in their studies. Since very different temperatures have been used in other studies we used -5°Cand 50°Cfor warming and cooling inlays. Inlay bonding procedure is more time-consuming than direct composite resins. Therefore, in this study we used some higher and lower temperatures than other studies to compensate temperature changes occurring during inlay bonding to achieve results that could be extended to in vivo conditions. To be sure of reaching of inlays to exact temperatures, the inlays were kept for 5 minutes at 5°C and 50°C. Since there were no references in the literature, this time was selected by authors' experience.

In vitro microleakage studies have not simulated pulpal pressure.^24^In this study, we used pulp pressure and a temperature device for simulating in vivo conditions to achieve more reliable results. Many studies have shown that heating composite resin increases free radical movement, increasing the degree of conversion, polymerization shrinkage and the resultant stress.^25,26^ Also, one of the effects of temperature on materials is thermal expansion and contraction. Warmed composite resin shrinks thermally when placed in the oral cavity temperature. On the other hand, higher temperatures can increase composite resin flowability and its adaptation to cavity walls. In theory, thermal expansion of cold composite resin due to warming under oral temperatures can reduce polymerization-induced gap but cooling increases viscosity and causes problems during restoration seating.^3^ Because of these different influences of heating and cooling, different studies have shown different results.

Minimum marginal gap in indirect restorations can be achieved with low film thickness. Adaptation of indirect restorations is one of the most important criteria of clinical success.^27^ In this study, cement film thickness was used for measurement of marginal integrity. A major concern about CAD/CAM restoration has been their pre-cementation marginal fit. However, improvements in CEREC hardware and software have led to improvements in marginal fit.^28^ Ellingsen and Fasbinder^29^ compared the pre-cementation fit of CERED2 crowns with CEREC3 crowns. CEREC3 had more marginal accuracy than CEREC2 in all areas. In the present study, like a study by Romao et al,^30^ the cement thickness was smaller in CAD/CAM inlays. This result may suggest that marginal integrity and accuracy of CAD/CAM system is not a concern anymore and these systems have acceptable accuracy for clinical use.

In this study, like that by Romao et al,^30^ no relation was found between microleakage and film thickness. It can be expected that better marginal adaptation can cause lower volume of resin cement and its resultant problems.^31^Resin cement polymerization-induced stress occurs in closed environments that can have negative effects on bond strength. Magnitude of this force depends on the ratio of bonded to non-bonded surfaces (C-factor). Therefore, in theory, it seems that there is an inverse relationship between the thickness of the resin and microleakage. Alester et al^32^ showed that a thin layer of resin (50–2700 µm) increases polymerization stress but on the other hand they reported that resin cement layer was too thin and even small deformation in the substrate could release polymerization shrinkage stress. It seems these two factors (increasing the polymerization shrinkage stress because of thin layer of resin cement and stress releasing due to substrate deformation) can affect each other and can be the reason for the results achieved in the present study.

According to the results of the present study, different temperatures of inlays and cement thickness had no significant effect on restoration microleakage. But in this study we changed the inlay temperature. Maybe changing resin cement temperature can directly affect microleakage. Further studies are required to investigate the effect of resin cement temperature on inlay microleakage.

## Conclusion

Within the limitations of this in vitro study, it can be concluded that heating or cooling of inlays had no significant effect on microleakage. CAD/CAM-made restorations had lower cement film thickness than laboratory-made restorations, resulting in a slight decrease in leakage, but this decrease was not statistically significant.
